# Calcium Channel Blockade Ameliorates Endoplasmic Reticulum Stress in the Hippocampus Induced by Amyloidopathy in the Entorhinal Cortex

**DOI:** 10.22037/ijpr.2019.111532.13216

**Published:** 2019

**Authors:** Azam Ghanbari-Maman, Forouzan Ghasemian-Roudsari, Shayan Aliakbari, Hamid Gholami Pourbadie, Fariba Khodagholi, Fatemeh Shaerzadeh, Mahtab Daftari

**Affiliations:** a *Department of Physiology and Pharmacology, Pasteur Institute of Iran, Tehran, Iran. *; b *Department of Biology, Faculty of Sciences, University of Zanjan, Zanjan, Iran.*; c *Neuroscience Research Center, Shahid Beheshti University of Medical Sciences, Tehran, Iran.*; d *Department of Neuroscience, College of Medicine and McKnight Brain Institute, University of Florida, Gainesville, FL, USA.*

**Keywords:** Alzheimer disease, Entorhinal cortex, Endoplasmic reticulum stress, Calcium channel blockers, Protein disulfide-isomerases

## Abstract

Entorhinal cortex (EC) is one of the first cerebral regions affected in Alzheimer’s disease (AD). The pathology propagates to neighboring cerebral regions through a prion-like mechanism. In AD, intracellular calcium dyshomeostasis is associated with endoplasmic reticulum (ER) stress. This study was designed to examine hippocampal ER stress following EC amyloidopathy. Aβ1-42 was bilaterally microinjected into the EC under stereotaxic surgery. Rats were daily treated with 30 μg of isradipine, nimodipine, or placebo over one week. Passive avoidance and novel object recognition (NOR) tasks were performed using shuttle box and NOR test, respectively. GRP78/BiP and CHOP levels were measured in the hippocampal dentate gyrus (DG) by western blot technique. The glutathione (GSH) level and PDI activity were also assessed in the hippocampus by colorimetric spectrophotometer. Aβ treated group developed passive avoidance and novel recognition memory deficit compared to the control group. However, treatment with calcium channel blockers reversed the impairment. BiP and CHOP level increased in the hippocampus following amyloidopathy in the EC. PDI activity and GSH level in the hippocampus decreased in the Aβ treated group, but calcium channel blockers restored them toward the control level. In conclusion, memory impairment due to EC amyloidopathy is associated with ER stress related bio-molecular changes in the hippocampus, and treatment with L-type calcium channel blockers may prevent the changes and ultimately improve cognitive performance.

## Introduction

The hippocampus, as a part of the limbic system, plays a critical role in the consolidation of information from short-term memory to long-term memory. One of the most consistent features of Alzheimer’s disease (AD) is hippocampal atrophy. Hippocampus is robustly affected in the early phase of the disease resulting in hippocampal/neocortical dissociation which leads failure of encoding of new information emanating from hippocampus (1). AD is a neurodegenerative disorder characterized by amyloidopathy in the cerebral regions involved in learning and memory (2). Aβ was produced through sequentially cleavage of amyloid precursor protein (APP) by beta and gamma secretases (3). Four main genes involved in AD are associated with cerebral Aβ accumulation (4). Previous studies have demonstrated that Aβ may activate neurotoxic cascades, that leads to synaptic disintegrity, mitochondrial dysfunction, oxidative stress, and neuronal loss (5). In AD, entorhinal-hippocampal circuit, which is involved in encoding spatial navigation and object recognition, is one of the earliest affected networks (6-8). Via perforant pathway, EC neurons project to all hippocampal sub-regions, including dentate gyrus (DG), CA3, CA1, and subiculum (9, 10). It has been shown that significant neuronal loss occurs in the EC layer II, the main source of projection to the DG, in the initial stage of AD (11). Via a prion like mechanism, AD may invade through interconnected cerebral regions (12, 13). Amyloidopathy induced molecular and neuronal changes initiating in the EC may spread through EC-hippocampal network (14). 

Aβ causes calcium dyshomeostasis through Ca^2+^ release from the intracellular sources such as endoplasmic reticulum (ER) or Ca^2+^ influx through ion channels including voltage gated calcium channels (15, 16). In this line, Aβ has been shown to increase high voltage-activated (HVA) calcium currents through N-type, P/Q-type, and also L-type calcium channels (17, 18). The ER is a major intracellular organelle involved in regulation of intracellular calcium homeostasis. ER-mitochondria Ca^2+^ transfer is involved in neuronal cell death due to Aβ (19). Accumulation of unfolded or misfolded proteins in the lumen of the ER results in a state named unfolded protein response (UPR) in order to halt protein translation, to degrade misfolded proteins, and to activate the signaling pathways leading to chaperones expression involved in protein folding. However, in AD, Ca^2+^ dyshomeostasis causes prolong accumulation of unfolded proteins that aims the response toward apoptosis (20, 21). Recent studies demonstrate that Aβ induces ER stress (22, 23). On the other hand, the elevated level of intracellular calcium increases the Aβ load which may induce a vicious cycle ultimately leading to neuronal cell death (24). UPR is initiated by binding the ER chaperone GRP78/BiP to the misfolded proteins. GRP78/Bip dissociation leads to autophosphorylation of double-stranded RNA-activated protein kinase-like ER kinase (PERK) and endoribonuclease IRE-1, and mobilization of transcription factor ATF-6 to the Golgi for activation (25). UPR also induces expression of other chaperones including HSP70, HSP90, and protein disulfide isomerase (PDI) to limit further accumulation of folding-defective proteins (26). PDI is a disulfide isomerase enzyme which helps to correct misfolded proteins properly by forming proper disulfide bonds. 

In AD, PDI is upregulated in the brain to prevent the accumulation of Aβ (27). Reactive oxygen species (ROS), as a byproduct of unfolded protein overloaded have been shown to be buffered by redox-signaling mediators such as glutathione (GSH) in ER stress conditions (28). 

We previously found that EC amyloidopathy induced aberrant Ca^2+^-dependent I_Ca+2_ inactivation (CDI) in the DG granule cells (29). Decreased calcium buﬀering capacity inside the neurons may result in increased level of calcium near the channel pore resulted in abnormal CDI. ER is a critical intracellular calcium buffering organelle which may be affected by the following microinjection of Aβ into the EC. Therefore, in this study, we first examined behavioral performance, and then measured ER stress-associated biomolecules in the hippocampus following EC amyloidopathy in order to examine whether EC amyloidopathy induces ER stress in the hippocampus, and treatment with calcium channel blockers could reverse the disturbance. 

## Experimental

Adult male Wistar rats (Pasteur Institute; Tehran, Iran) weighing between 220 ± 20 g at the time of surgery were used. The animals were housed four per standard cage, in a colony room with a 12/12 h light-dark cycle (7:00–19:00 lights on) at 22 ± 1 °C. Commercial rodent pellets and tap water were available *ad libitum*. They were allowed to adapt to the laboratory conditions for at least one week before surgery. The rats were handled about 5 m each day prior to behavioral test. Ten animals were used in each group of the experiments. A total number of 40 animals were used in the experiments. All procedures were carried out in accordance with the institutional guidelines for animal care and use.

*Drugs and Surgery*

The drugs used in the study were ketamine and xylazine (Alfasan Chemical Co, Woerden, Holland) for animal anesthesia, Aβ1-42 (Tocris, UK), isradipine, and nimodipine (Tocris, UK). Aβ was dissolved in 0.1 M phosphate-buffered saline (PBS; pH 7.4) and then kept at -70 °C until use. One week before the beginning of the behavioral experiments, the animals were anesthetized and Aβ or vehicle (DMSO) were bilaterally microinjected into the entorhinal cortex under the stereotaxic surgery (AP: -5.05, L: ± 6.6 and DV: -8.2) according to the Paxinos and Watson Atlas (30). Injections were done over 2 min using a 30-gauge blunt-tipped needle attached to a 5-μL Hamilton syringe, and to avoid fluid back flow the needle remained in place for an additional 1 min. After entorhinal injection, a cannula (8 mm, 23 gauge) was implanted 1 mm above the right ventricle (AP: -0.96, L: 1.8, DV: -3.4) and then secured to the skull using dental cement. Over one week, the rats were treated daily with i.c.v. microinjection of nimodipine, isradipine (both 30 μg/μL, Tocris, UK), or vehicle (DMSO, Sigma-Aldrich, 

USA). 

The animals were divided into four groups (n = 10 each group): (a) Control group; given single microinjection of PBS (2 μL) into the entorhinal cortex, and daily DMSO (2 μL) microinjection into the right ventricle over one week. (b) DMSO-Aβ group; treated by Aβ (1 µg/2 μL) in the entorhinal cortex, and treated by daily microinjection of DMSO (2 μL) into the right ventricle for one week. (c) Aβ + ISR group; received Aβ (1 µg/2 μL) in the entorhinal cortex and daily treated by isradipine (30 µg/2 μL) for one week. (d) Aβ + NIM; received single microinjection of Aβ into the entorhinal cortex and daily microinjection of nimodipine (30 µg/2 µL) into the lateral ventricle. 

*Behavioral testing*

*Passive avoidance learning and memory*

The Shuttle box consisted of two compartments, light and dark compartment (20 cm × 20 cm × 30 cm) which were divided by a guillotine door (7 cm × 9 cm). The floor composes of stainless steel grids (2.5 mm in diameter) with one-centimeter intervals and connected to an insulated stimulator. In order to habituate to the apparatus, each animal was put in the light chamber and 10 sec later the guillotine door was opened to allow the rat to enter the dark chamber. When the animal entered the dark compartment, the door was closed, and the rat was returned back to its home cage. After 30 min, the rat was placed in the light chamber and 10 sec later the door was opened and when the animal entered the dark compartment, the door was closed and a foot shock (50 Hz, 1.5 mA for 1.5 sec duration) was applied. After 10 sec, the rat was taken and returned to its cage. Two min later, the rats were allowed to repeat the task and the training was completed when they avoided entering the dark compartment for 120 sec. After 24 h, the rats were again placed in the light compartment and after 10 sec the door was opened, and the step-through latency and the time spent in the dark compartment were recorded for 10 min.

*Novel object recognition test*

The Novel Object Recognition (NOR) test took place in a wooden open field box (40 × 40 × 60 cm). This test consists of the habituation phase, the familiarization phase, and the test phase. On the first day, the rats were habituated to the apparatus in which they were placed in the empty open field for 5 min. Twenty-four h later, the rats were allowed to explore two identical objects over 5 min. Twenty-four h after the training session, one of the object was replaced with a new object with distinctive shape and the rats were allowed to explore the open field for 5 min in the presence of two objects; the familiar object exploration (FOE) and a novel object exploration (NOE). Between trials, the objects were washed with 10% ethanol solution. The object exploration was measured using two stop watches to record the time spent exploring the objects during the experimental sessions. 

*Brain collection*

After completion of the experimental sessions, each animal was decapitated and the right and left hippocampi were immediately removed and washed in the PBS. Then, the hippocampi were deep-frozen and stored at -80 °C until use. For BiP and CHOP protein level measurement, the animals were decapitated and their brains were horizontally sliced (500 µm) by vibroslicer (Campden Instruments, UK). Then, the dentate gyrus tissues were separated under loop microscope and homogenized in the lysis buffer containing a protease inhibitor cocktail and stored at -80 °C until use.

*Western Blot Analysis*

Protein concentrations of hippocampal and DG lysates were quantified using Bradford method to guarantee equal loading for assaying by electrophoresis and GSH assay (31). They were lysed in lysis buffer containing complete protease inhibitor cocktail (Roche, Germany). For western blotting, 50 µg of DG total protein was electrophoresed in 12% SDS-PAGE gels, transferred to PVDF membranes and probed with anti-CHOP, anti BiP (1/1000 dilution, Cell Signaling Technology, Beverly, MA, USA), and anti β-actin (1/1000 dilution, Abcam, Cambridge, UK) as internal control. Electrochemiluminescence (ECL) reagents (Amersham Bioscience, USA) were used to detect immunoreactive polypeptides and then the results were quantified using densitometric scan of the films. Data analysis was done using ImageJ software (National Institute of Health).

*Determination of GSH activity*

Glutathione reduction was measured using the Ellman’s methods (32). Briefly, 100 μL of Ellman′s reagent (DTNB, 0.1 M) was mixed with 200 μL of hippocampus lysate and 0.25 mL sodium phosphate buffer (0.1 M). The amount of glutathione was measured by spectrophotometer method at 412 nm and the results were expressed as GSH/μg protein. 

*PDI assay*

PDI activity was determined by measuring reduction of insulin in the presence of dithiothreitol (DTT) as a reducing agent. The cocktail contained 100 mM potassium buffer, 0.2 mM EDTA (pH 7.0), 0.15 mM insulin, and 1 mM DTT. Protein sample was added to solution and the polymerization of reduced insulin chains was traced at 650 nm (33). 

*Data Analysis*

The data were expressed as mean ± standard error of the mean and processed by GraphPad Prism 5.0. Results were statistically evaluated by One-way analysis of variance (ANOVA). Where F value was significant, post-hoc analysis (Tukey test) was performed. Object recognition test was analyzed using unpaired *t*-test. Differences with *p* < 0.05 between the groups were considered statistically significant.

## Results

Microinjection of Aβ into the entorhinal cortex resulted in cognitive impairment which was reversed by calcium channel blockers 

**Figure 1:**
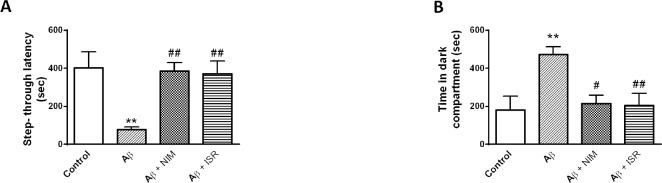
Calcium channel blockers attenuated the deteriorative effect of Aβ on passive avoidance learning and memory. (A) Aβ treated rats showed less latency to enter the dark compartment (step through latency) compared to the control group and treatment with isradipine or nimodipine could increase the latency toward the control level. (B) Microinjection of Aβ into the entorhinal cortex increased the time spent in the dark compartment in contrast to the control group, and treatment by isradipine or nimodipine reverse the deteriorative effect of Aβ. Data are expressed as mean ± SEM. **p < 0.01 compared to the control and #*p *< 0.05, ##*p *< 0.01 compared with the Aβ group

As shown in Figure 1, Aβ microinjection into the entorhinal cortex impaired passive avoidance learning and memory. The latency to cross the dark compartment in the Aβ treated rats was significantly reduced in contrast to the control group (*p* < 0.01); however, this effect was blocked by daily microinjection of 30 μg nimodipine or isradipine (Figure 1A). Also, Aβ group spent more time in the dark compartment compared to the control group (*p *< 0.01), and i.c.v. microinjection of isradipine or nimodipine increased the time toward the control level (Figure 1B).

**Figure 2:**
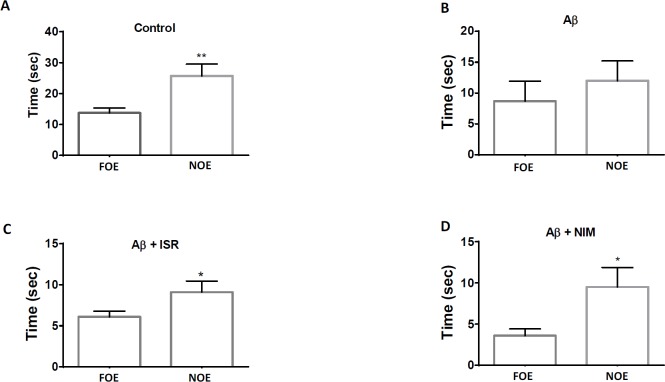
Calcium channel blockers restore the novel object recognition performance in AD model of rats. (A) Control group spent more time to explore the novel object (NOE) compared to the familiar object. (B) However, no difference of exploration time between familiar and novel object was observed in Aβ treated rats. (C) and (D) Treatment of AD rats with isradipine or nimodipine could restore the NOR task. Data are expressed as mean ± SEM

As shown in Figure 2, the time of exploration of novel object (NOE) in the control group was greater than that of the familiar object (FOE). However, no difference was observed between NOE and FOE in Aβ treated rats. Intriguingly, the difference between NOE and FOE in Aβ treated rats was restored following treatment with isradipine or nimodipine.

*Isradipine reversed elevated levels of BiP and CHOP in the dentate gyrus following EC amyloidopathy*

**Figure 3:**
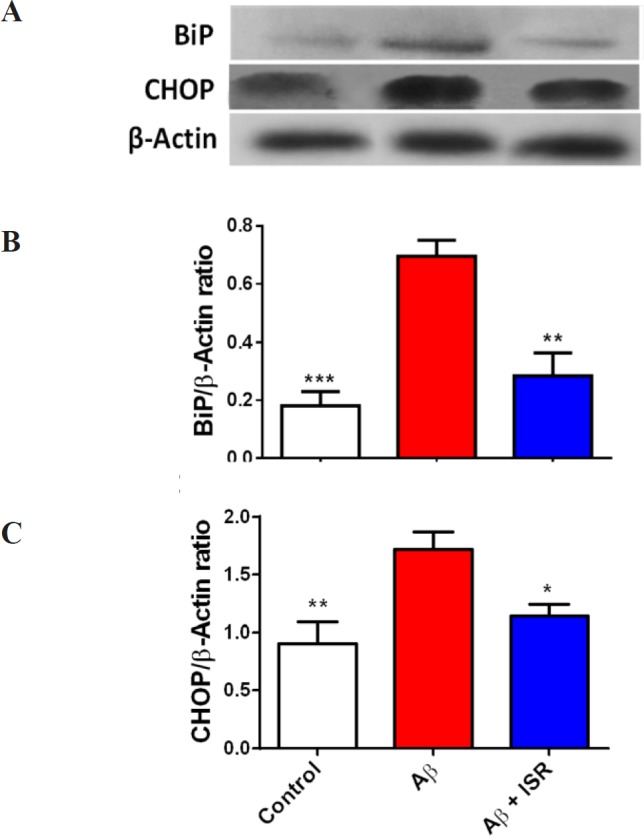
Amyloidopathy in the entorhinal cortex induced alteration of ER stress associated biomolecules in the hippocampal dentate gyrus. (A) The representative blots of BiP and CHOP obtained by western blot. (B) Microinjection of Aβ into the entorhinal cortex resulted in higher BiP level in the dentate gyrus. However, daily microinjection of isradipine restored it to the control level. (C) CHOP level in the hippocampus was increased following amyloidopathy in the entorhinal cortex and treatment with isradipine could prevent its rise in the DG of Aβ treated rats. Data are expressed as mean ± SEM. ^*^p < 0.05, ^**^p < 0.01 and ^***^p < 0.001 compared with the Aβ treated group

Soluble Aβ has been shown to elicit ER stress-induced unfolded protein response (UPR) and Ca^2+^ dyshomeostasis. For this, BiP and CHOP as two main ER stress markers were assessed in this study. As shown in Figure 3A, protein level of BiP was increased in the DG following Aβ microinjection into the DG. However, in the animals that received daily microinjection of isradipine (30 μg), its level was restored to the normal level. Also, CHOP was upregulated in the DG following entorhinal amyloidopathy, and treatment with isradipine decreased the CHOP protein level.

*Decreased PDI activity in the hippocampus following entorhinal amyloidopathy was restored by calcium channel blockers*

**Figure 4:**
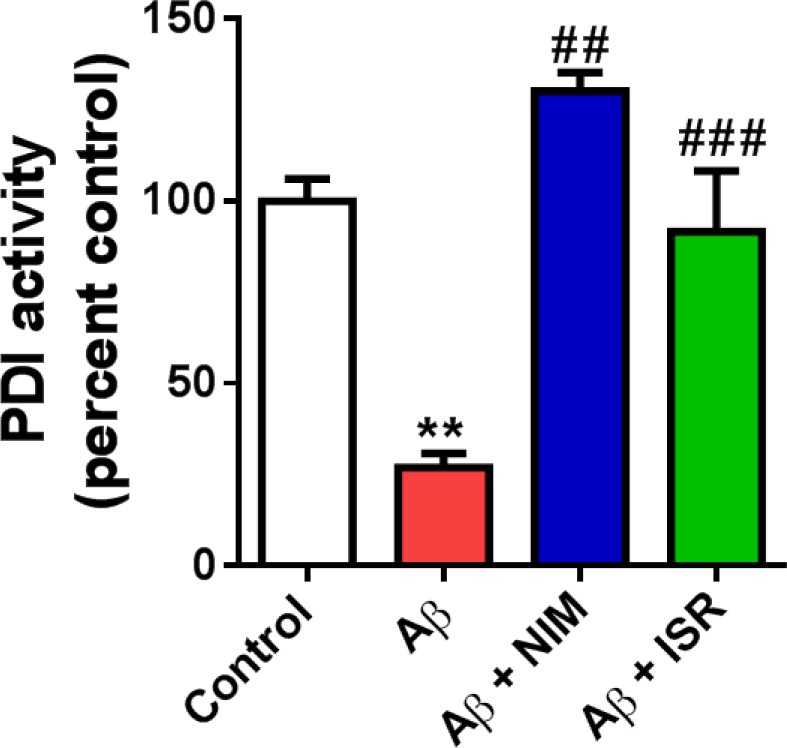
Decreased PDI activity in the hippocampus following microinjection of Aβ into the entorhinal cortex was reversed by calcium channel blockers, isradipine or nimodipine. The PDI-catalyzed reduction of insulin in the presence of DTT was measured to assay PDI activity (n = 4). ^**^p < 0.01 compared to the control group and ^##^*p* < 0.01, ^###^*p* < 0.001 compared to the Aβ group

PDI is an ER resident enzyme that catalyzes the formation and breakage of disulfide bonds in newly synthesized proteins in order to form proper folding of protein structures. As shown in Figure 4, PDI activity decreased following EC amyloidopathy compared to the control group (*p* < 0.01). However, treatment with isradipine or nimodipine restored the enzyme activity in hippocampus compared to the Aβ treated group (*p* < 0.01).

*EC amyloidopathy decreased GSH level in the hippocampus and treatment with calcium channel blockers reversed the Aβ effect on GSH level*

**Figure 5:**
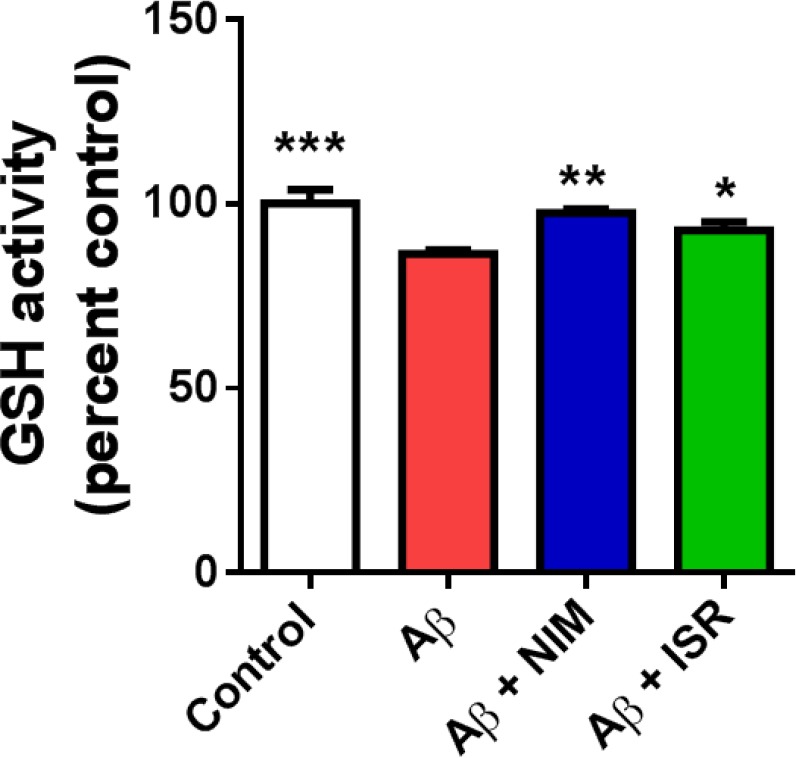
Effect of intra-EC microinjection of Aβ on GSH level in the hippocampus. Microinjection of Aβ into the EC decreased the GSH level in the hippocampus compared to the control group. However, treatment with isradipine or nimodipine could restore the GSH level in the Aβ treated group (n = 4-5 in all group). (^*^p < 0.05, ^**^p < 0.01 and ^***^p < 0.001 compared with the Aβ

Accumulating evidence suggests the interrelation of ER stress and ROS with redox signaling mediators such as PDI-ER oxidoreductin (ERO)-1, glutathione (GSH)/glutathione disulphide (GSSG), and calcium (28). Microinjection of Aβ into the EC significantly (*p* < 0.001) decreased the GSH level in the hippocampus compared to the control group, whereas treatment with nimodipine or isradipine (*p* < 0.01, *p* < 0.05) prevented the decrease of GSH in the hippocampus following EC amyloidopathy (Figure 5).

## Discussion

The present study was conducted to assay ER stress associated molecules in the hippocampus following Aβ microinjection into the entorhinal cortex. In a previous work, we found that EC amyloidopathy decreased the amplitude of calcium current and increased Ca^2+^-dependent I_Ca+2_ inactivation (CDI) in the dentate gyrus granule cells (29). Increased intracellular level of Ca^2+^ or decreased calcium buﬀering proteins inside the neurons may increase calcium concentration near the channel pore and then increase CDI. We previously found that no alteration of calbindin, as a main buffering protein, was happened in the dentate gyrus following EC amyloidopathy. Abnormal Ca^+2^ current could be associated to ER stress and dysfunction due to its critical role in intracellular calcium buffering. Therefore, in this study, we examined ER stress related biomolecules in the hippocampus following EC amyloidopathy. We found that BiP and CHOP protein level, glutathione level and PDI activity changed in the hippocampus following amyloidopathy in the EC and calcium channel blockers prevent the changes. 

The EC-hippocampal network which is involved in spatial navigation and novel object recognition is affected in the initial phases of AD (8, 34). EC lesions induce re-circuiting in the dentate gyrus which may change the molecular profile and electrophysiological properties in the dentate gyrus (14, 35 and 36). In this line, we previously found that microinjection of Aβ into the EC decelerated spatial learning and memory induced imbalance of inhibitory and excitatory transmission in favor of GABAergic currents and prompted abnormal intrinsic electrophysiological properties in the DG granule cells (34, 37 and 38). Consistently, Harris *et al.* reported that APP/Aβ expression in the EC caused cognitive and behavioral abnormalities such as spatial learning and memory deficits relevant to synaptic dysfunctions. They also reported that molecular expression profile is changed in the dentate gyrus following EC amyloidopathy (14). Aβ may also affect microglial activity and consequently alter DG electrophysiological properties (39). In this regard, we observed that microinjection of Aβ into the EC induced abnormal calcium current with prominent CDI in the dentate gyrus (29). This phenomenon is associated with the cellular Ca^2+^ buffering capacity. Here, we found that ER is affected by EC amyloidopathy. Neuronal cells have elaborative ER that extends from the cell body to the axons and even dendrites which provides the ER with precise control of Ca^2+^ level and signaling and consequently neuronal excitability (40). ER controls the intracellular Ca^2+^ level by uptaking Ca^2+^ from the cytosol through sarcoplasmic/endoplasmic reticulum Ca^2+^ATPase or releasing Ca^2+^ via IP3Rs/ryanodine receptors (41). Aβ is believed to render neurons more susceptible to excitotoxicity through persistent rise of intracellular Ca^2+^ levels (42). Multiple studies have shown the role of L-type Ca^2+^ channels in Aβ-induced neuronal toxicity in both in vitro and in vivo (38, 43, 44). Large conductance Ca^2+^ activated K^+^ channel (BK channel) has been also reported to be involved in neurodegenerative disorders (45). It has been shown that alteration of K^+^ ion channel activity may alter neuronal function (46). Resende *et al.* demonstrated that soluble Aβ could induce cell death through intracellular calcium dyshomeostasis due to Ca^2+^ release from ER (15). 

The ER stress response is considered as an important process in the etiology of AD. Under stress conditions, the cells evade serious damage by activating adaptive response pathways including UPR. The UPR is initiated by the binding of the ER chaperone GRP78/BiP to the misfolded proteins. Under normal conditions, GRP78/Bip binds to a three signaling mediators including double-stranded RNA-activated protein kinase-like ER kinase (PERK), transcription factor (ATF-6), and endoribonuclease (IRE-1) to form inactive complex (23, 47). Dissociation from GRP78/Bip seems to activate other three key signal transducers. Both PERK and IRE1 pathways result in CHOP expression, a chaperon that induces apoptosis (41). Two important factors involved in ER stress are caspase 12 and calpain 2. Previously, we showed that EC amyloidopathy led to caspase 12 activation in the dentate gyrus, which functions as an ER-specific caspase (34). Finding novel therapeutic agents to treat AD is a major interest (48, 49). Our findings provide evidence that isradipine and nimodipine protect neurons against apoptosis under conditions associated with ER stress. In agreement, multiple studies have demonstrated that calcium channel blockers reduce apoptosis through regulation of ER stress (50, 51). Our results revealed that EC amyloidopathy upregulated CHOP and BIP in the dentate gyrus while PDI activity and GSH level decreased in the dentate gyrus. However, daily microinjection of calcium channel blocker into the cerebral ventricle prevented the change of these ER stress related biomolecules. Consistently, it has been reported that both BiP and CHOP expression increased in experimental models of AD (52). Additionally, elevation of BiP has been reported in the hippocampus and temporal cortex of AD patients (53). On the contrary, Katayama et al. reported a significant decrease in BiP expression in a cell model of AD (54). The controversial result may be due to using different experimental design and models. 

It has been shown that expression of CHOP and its subsequent ROS accumulation and cell death occurred in ER stress (28, 55). On the other hand, BiP has been shown to block CHOP expression and reduces ER-stress induced apoptosis (23). CHOP affects the activity of the PDI by inducing ER oxidoreductin 1 (ERO1α). In the processes of protein folding, ERO1α helps transferring electrons from PDI to molecular oxygen to generate disulfide bonds in proteins leading to hydrogen peroxide (H_2_,O_2_) production (28). Glutathione antioxidants (GSH) are required to remove H_2_O_2_. Therefore, there is an association between GSH and PDI and CHOP in acute and chronic ER stress. We observed that the level of PDI and GSH decreased in the Aβ treated group which may be due to their consumption in response to UPR and increased ROS production, respectively. Consistently, it has been shown that fibrillar Aβ decreases PDI activity continuously over ten days in the hippocampus (26). In contrary increased PDI expression has been reported in the AD and old healthy subjects (56). Decreased PDI activity observed in our work may reflect its low content due to its consumption. To support this view, high levels of the Aβ-PDI complex is reported in the cerebrospinal fluid of AD patients which indicates PDI removal form the cells and brain parenchyma. Also, decreased PDI activity may arise from s-nitrosylation due to increase NO production following Aβ treatment (27, 57). Therefore, increasing PDI activity may be considered as a protective strategy of neuronal cells against Aβ-induced ER stress. Also, we showed that blocking the calcium channel could restore PDI activity in Aβ group. In this line, Tohda *et al.* reported that pharmacological activation of the PDI enzyme could decrease Aβ load in the hippocampus and improve object recognition in AD mice model (58). 

In conclusion, amyloidopathy in the entorhinal cortex resulted in ER stress associated molecular changes in DG, and calcium channel blockers prevented the changes. In the early stages of AD, the molecular alteration related to the ER stress may cause calcium dyshomeostasis in the neighboring regions, leading to AD propagation from EC to DG and other cerebral regions. However, further investigation will be required to elucidate the contribution of ER stress in AD progression. 
